# The impact of ketogenic diet on some metabolic and non‐metabolic diseases: Evidence from human and animal model experiments

**DOI:** 10.1002/fsn3.3873

**Published:** 2024-01-08

**Authors:** Yaseen Galali, Salih M. S. Zebari, Ahmed Aj. Jabbar, Holem Hashm Balaky, Bashdar Abuzed Sadee, Hamed Hassanzadeh

**Affiliations:** ^1^ Food Technology Department College of Agricultural Engineering Sciences, Salahaddin University‐Erbil Erbil Iraq; ^2^ Department of Nutrition and Dietetics Cihan University‐Erbil Erbil Iraq; ^3^ Animal Resource Department College of Agricultural Engineering Sciences, Salahaddin University‐Erbil Erbil Iraq; ^4^ Department of Medical Laboratory Technology Erbil Technical Health and Medical College, Erbil Polytechnic University Erbil Iraq; ^5^ General Science Department, Faculty of Education Soran University Erbil Iraq; ^6^ Mergasor Technical Institute Erbil Polytechnic University Erbil Iraq; ^7^ Department of Food Science and Technology, Faculty of Para‐veterinary Ilam University Ilam Iran

**Keywords:** cardiovascular disease, diabetes and weight loss, keto diet, metabolic and non‐metabolic diseases

## Abstract

The ketogenic diet (KD) is recognized as minimum carbohydrate and maximum fat intakes, which leads to ketosis stimulation, a state that is thought to metabolize fat more than carbohydrates for energy supply. KD has gained more interest in recent years and is for many purposes, including weight loss and managing serious diseases like type 2 diabetes. On the other hand, many believe that KD has safety issues and are uncertain about the health drawbacks. Thus, the outcomes of the effect of KD on metabolic and non‐metabolic disease remain disputable. The current narrative review aims to evaluate the effect of KD on several diseases concerning the human health. To our best knowledge, the first report aims to investigate the efficacy of KD on multiple human health issues including type 2 diabetes and weight loss, cardiovascular disease, kidney failure and hypertension, non‐alcoholic fatty liver, mental problem, oral health, libido, and osteoporosis. The literature searches were performed in Databases, PubMed, Scopus, and web of Science looking for both animal and human model designs. The results heterogeneity seems to be explained by differences in diet composition and duration. Also, the available findings may show that proper control of carbohydrates, a significant reduction in glycemic control and glycated hemoglobin, and weight loss by KD can be an approach to improve diabetes and obesity, hypertension, non‐alcoholic fatty liver, PCOS, libido, oral health, and mental problem if isocaloric is considered. However, for some other diseases like cardiovascular disease and osteoporosis, more robust data are needed. Therefore, there is robust data to support the notion that KD can be effective for some metabolic and non‐metabolic diseases but not for all of them. So they have to be followed cautiously and under the supervision of health professionals.

## Graphical abstract

The ketogenic diet (KD) is recognized as minimum carbohydrate and maximum fat intake, which leads to ketosis stimulation, a state that is thought to metabolize fat more than carbohydrates for energy supply. KD has gained more interest in recent years and is for many purposes, including weight loss and managing serious diseases like diabetes.

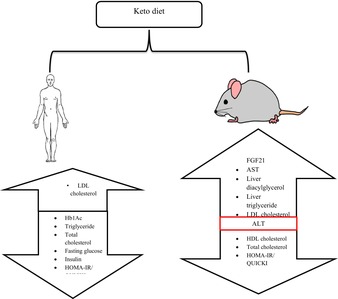

## 1 INTRODUCTION

Ketogenic diet or keto diet (KD) is known as a low‐carbohydrate (5%–10%), medium‐protein (15%–20%), and high‐fat (75%) diet (Sukkar et al., 2021). The diet was initially invented in the early twentieth century and used to treat children with refractory epilepsy. Nowadays, it is one of the most widely followed diets for weight loss as well as some other metabolic disorders as a nutritional intervention therapy (Hermanussen et al., 2018). Ketogenesis commences with fatty acid (FA) lipolysis into free FA that are taken from adipose tissue to hepatocyte and further converted into acetyl coenzyme A (CoA). Under limited sugar conditions, acetyl coenzyme A is converted by activity of thiolase enzyme to acetoacetyl CoA. β‐hydroxy‐β‐methylglutaryl CoA is then synthesized from acetoacetyl CoA. At the final stage, three main ketone bodies are produced by the mitochondria which are acetone, acetoacetate, and 3‐β‐hydroxybutyrate. Acetone is eliminated by the exhale via lungs or by urination. Coincidentally, 3‐β‐hydroxybutyrate and acetoacetate diffuse into the blood circulation but not reaching liver. Ketolysis has to happen to generate ketone bodies. During this process, the two aforementioned ketone bodies circulating in the blood are changed back through succinyl CoA: 3‐oxoacid CoA transferase into acetyl CoA and then acetyl CoA acetyltransferase. Next, through the Krebs cycle, Acetyl CoA is further metabolized to generate 22 ATP/molecules (Weber et al., 2020).

Based on the diet composition, there are a number of KD classifications including classical KD, medium‐chain triglyceride KD, low glycemic index treatment KD, Atkins KD, and modified Atkins KD. They are all entitled to KD; however, their protein, carbohydrate, and fat content are different. However, others are just classified into one single classification (Trimboli et al., 2020). In recent years, the evidence has increasingly accumulated regarding using different types of low‐carb diet (LCD) mainly KD as a therapy for a number of diseases. Particularly using very low‐calorie KD is a promising approach for metabolic diseases including obesity but this should be personalized and side effects should be taken into account (Barrea et al., 2022). As mentioned earlier, the diet has been used for decades to treat epilepsy. But recently, the interest in using LCD and/or KD as a therapeutic interventional option for many metabolic diseases has significantly increased. Moreover, it has an effective potent in mitigating a number of metabolic diseases such as type 2 diabetes and obesity (Tay et al., 2014), cardiovascular disease (Cicero et al., 2015), renal function and hypertension (Omozee & Osamuyimen, 2019), polycystic ovary syndrome (Mavropoulos et al., 2005), and others. On the other hand, many believe that KD has safety issues and is uncertain about the drawbacks. The outcomes of the effect of KD on metabolic and non‐metabolic disease remain debatable (Zhang et al., 2021).

Therefore, this review highlights the impact (negative and positive) of following KD on some metabolic and non‐metabolic diseases including cardiovascular diseases, diabetes, hypertension, PCOS, Libido, and skeletal muscle based on human and animal model experiments.

## 2 METHODS

### 2.1 Study selection

For study selection, a systematic literature search was conducted in PubMed, Scopus, and Web of Science database according to the PRISMA guidelines (Moher et al., 2010). The KD defined is the diet with no more than 50 g carbohydrates daily. *Ketogenic* OR *ketogenic diet* OR *keto diet* OR *ketogenous diet* OR *ketotic diet* OR *very low‐carb diet*. The researches were assessed by assessing the title and the summary (abstract) from September 2022 to September 2023. The potential eligible studies were retrieved to evaluate for inclusion criteria.

### 2.2 Study eligibility criteria

For eligibility criteria, all human and animal model trial publications using KD in English language during our review period were included. *Keto diets* were used as keywords in order to avoid confusion. We included studies conducted on animal and human models, guidelines, case reports, reviews, and meta‐analyses were also included focusing on *Diabetes Mellitus* OR *diabetes* AND *Cardiovascular disease* OR *Kidney failure* OR *Hypertension* OR *Tooth decay* OR *Osteoporosis* OR *Fatty liver*. The included studies were evaluated individually, and the risk of bias was assessed for each study. Studies using the same results or duplicate studies were excluded.

### 2.3 The data extraction

**FIGURE 1:**
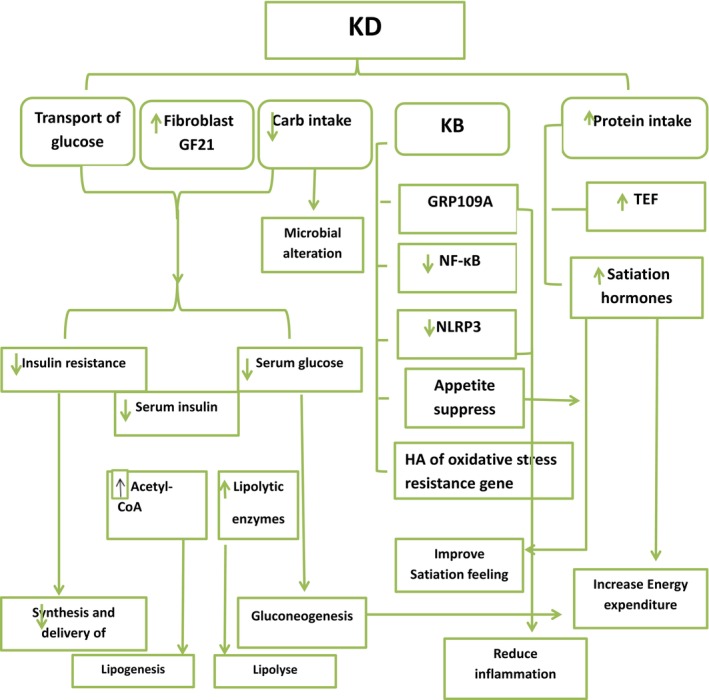
Elucidates the impact of KD on overall metabolic disorders including non‐alcoholic fatty liver, type 2 diabetes, obesity, and polycystic ovary syndrome through reducing inflammation, increasing energy expenditure and satiation, and reducing weight. KB, ketone bodies; TEF, thermo‐effect of food.

The data extraction for the studies was done for the studies by selecting interested measures (publication year, study duration, KD composition, and BMI; Figure 1). The impact of KD is on some clinical parameters (systolic and diastolic blood pressure, BMI and weight, lipid profile, (triglyceride, total cholesterol, LDL, and HDL)), glycemic parameters (fasting insulin, fasting glucose, HbA1c, HOMA‐IR, skeletal structure, PCOS (Hyperinsulinemia, hyperandrogenism)), mental and psychiatric parameters (mood stabilization and anxiety score), hepatic profile liver enzyme levels as alanine aminotransferase (ALT), and aspartate aminotransferase (AST).

## 3 RESULTS

### 3.1 The impact of KD on metabolic diseases

#### 3.1.1 Type 2 diabetes and weight loss

Type 2 diabetes is one of the most common metabolic diseases, and the number of type 2 diabetic people is increasing worldwide (Ong et al., 2023). According to the data, approximately 425 million people are diagnosed with diabetes globally. In the case of continuing this trend, it is expected that this number would increase to 629 million ranging from 20 to 79 years old (Bolla et al., 2019). One of the key managements of the type 2 diabetes is nutrition and healthy diet. During the past decades, several types of diets have been recommended by nutritionists, dieticians, and health professionals. Among these diets, KD (LCD) is gaining popularity due to its impact on losing weight and managing other metabolic problems like type 2 diabetes.

Several studies have shown that LC including KD can be useful in managing DM (Castro et al., 2018). Studies have shown that following KD can be useful in terms of metabolic disease through controlling insulin, reducing weight, and increasing satiation (Table 1). There are a number of studies showing the comparison between LC and/or KD and other diets (Goldstein et al., 2011; Hussain et al., 2012; Westman et al., 2008). The majority of the studies have pointed out that LC and KD can be useful in reducing the risks of type 2 diabetes. Different mechanisms have been proposed where KD can be a promising diet to reduce the fasting blood glucose and glycated hemoglobin. Furthermore, KD can substantially lose body weight and fat and consequently improve insulin resistance.

**TABLE 1:** Summary of included studies related to type 2 diabetes.

No	References	Design/sample	Intervention/diet	Duration of intervention	Major findings
1	Dashti et al. (2007)	*n* = 64 BMI <30 Obese healthy cases with high blood sugar (31) and normal blood sugar (33)	Low‐carb KD: 20 g/day of carb, ~80 g/day of protein	56 weeks	↘Bodyweight, ↘body mass index, ↘the level of blood glucose, ↘total cholesterol, ↘LDL‐cholesterol, ↘triglycerides, and ↘urea, while ↗HDL cholesterol (*p* < .0001)
2	Westman et al. (2008)	*n* = 84 BMI 27–50 kg/m^2^ Obese diabetic cases	Low‐carb KD (*n* = 38): 20 g/day of carb, low glycemic index (*n* = 46): 500 kcal less carb calories than daily carb intake	24 weeks	↘HbA1c, ↘fasting glucose, ↘fasting insulin, and ↘weight loss, and HDL. Diabetic drugs reduced in 95.2% of LCKD versus 62% of LGID cases (*p* < .01)
3	Goldstein et al. (2011)	*n* = 52 BMI 30–39.9 kg/m^2^ Type 2 diabetic cases	Stage 1 (4 weeks): diet with 80% of their caloric requirements based on DASH (the dietary approach to stop hypertension) Stage 2 (13 weeks): Atkins diet (25–40 g/day of carb) or standard recommended American Diabetes Association calorie‐restricted diet (g 10%–20%) of the daily energy intake from protein and the other 80% from fat Stage 3 (13–52 weeks): same diet, but the ATK and ADA groups had diets with fewer carbohydrates than the DASH diet	12–52 weeks	↔Weight, ↘systolic blood pressure, ↘diastolic blood pressure (mmHg), ↘HbA1c (%), ↔fasting glucose (mg/dL), ↘total cholesterol (mg/dL), ↘triglyceride (mg/dL), ↗HDL cholesterol (mg/dL), ↔microalbumin (mg/L), ↔urea (mg/dL)
4	Hussain et al. (2012)	*n* = 363 BMI <25 kg/m^2^ Obese and overweight cases including 102 cases were type 2 diabetes	Low‐carb diet and low‐carb KD were instructed: with initial goal of ~20 g/day of carb intake	24 weeks	↘Body weight, ↘blood glucose, and ↘waist circumference of the diabetic, ↘HbA1c, ↘LDL, ↘total cholesterol, ↘triglycerides, ↗ urea, and ↗ HDL of diabetic and non‐diabetic cases (*p* < .0001). While ↘uric acid and ↘ creatinine noticed in LCKD group
5	Wycherley et al. (2016)	*n* = 115 BMI 34.6 ± 4.3 kg/m^2^ Obese adults with type 2 diabetes	Low‐carb KD (57): 14% carbohydrate (<50 g/day), 28% of protein, and 58% of total fat (35% monounsaturated fat and 13% polyunsaturated fat) High‐carb diet (58): 53% of carbohydrate (emphasis on low glycemic index foods), 17% of protein, and <30% of total fat (15% monounsaturated fat and 9% polyunsaturated fat)	52 weeks	In both groups, ↔ HbA1c, ↔ FPG ↔ BMI, ↔ WC Compared to high‐carb, low‐carb KD group, ↔ MAGE (*p* = .09), ↘ CONGA‐1 (*p* = .003), ↘ CONGA‐4 (*p* = .02), ↔ time in euglycemia, ↔ time in hypoglycemia, ↘ medication (*p* = .02), ↘ TAG (*p* = .001), ↗ HDL‐c (*p* = .002)
6	Goday et al. (2016)	*n* = 89 BMI 33.1 ± 1.6 kg/m^2^ Obese cases with type 2 diabetes	Low‐carb KD (45): 600–800 kcal/day, carb <50 g/day, protein 0.8–1.2 g/kg, fat (10 g of olive oil) Hypocaloric diet (44): 500–1000 kcal, CHO 45%–60%, protein 10%–20%, fat <30%	13 weeks	Compared to the end of stage 1, in both groups, ↘ HbA1c (*p* < .0001), ↔ FBG, ↔ weight, ↗ TAG (*p* = .027), ↘ T‐Chol (*p* = .038), ↗ HDL‐c (*p* = .0026). Decreased oral antidiabetic medication (*p* = .0267)
7	Saslow, Mason, et al. (2017)	*n* = 25 BMI ≥25 kg/m^2^ Obese cases with type 2 diabetes	Low‐carb KD (12): 25–50 g/day of carbohydrates, ~20% of calories from carbohydrates Control diet (13): More than 50% of calories from carbohydrates	32 weeks	Compared to the control group, VLCKD had ↘ HbA1c (*p* = .002), ↘ weight (*p* < .001), ↘ TAG (*p* = .01), ↔ HDL‐c, ↔ LDL‐c
8	Saslow, Daubenmier, et al. (2017)	*n* = 34 BMI > 25 kg/m^2^ Obese cases with type 2 diabetes	Low‐carb KD (16): 20–50 g/day of carbohydrate Moderate carbohydrate, calorie‐restricted, low‐fat (MCCR) diet (18): 500 kcal reduced/day, 45%–50% of daily calories from carb, low fat	52 weeks	Compared to baseline, LCKD ↘ HbA1c (*p* < .007), ↔ fasting insulin, ↔ HOMA index ↘ medication (SU and DDP‐4 inhibitors, *p* = .005, metformin *p* = .08), ↘ weight (*p* = .001)
9	Hallberg et al. (2018)	*n* = 349 (262 in intervention) BMI 40.4 ± 8.8 kg/m^2^ Obese cases with type 2 diabetes	Restricted carb diet: >30 g/day of carb., 1.5 g/kg of daily protein intake	52 weeks	↘ HbA1c (*p* < .0001), ↘ FPG (*p* < .0001), ↘ fasting insulin (*p* < .0001), ↘ HOMA index (*p* < .0001), ↘ diabetes medication, except metformin (↗) and GLP‐1 (↔) ↘ weight (*p* < .0001), ↘ TAG (*p* < .0001), ↗ HDL‐c (*p* < .0001), ↘ LDL‐c (*p* < .0001), ↘ ALT (*p* < .0001), AST (*p* < .0001)
10	Barbosa‐Yañez et al. (2018)	*n* = 36 BMI 35.0 ± 5.0 kg/m^2^ Obese cases with type 2 diabetes	Low‐carb KD (16): ≤40 g/day of 5%–10% carb, 20%–30% protein, and 60%–70% fat Low‐fat diet (20):1000–1200 kcal/day from diet with 50% carb, 20% protein, <30% fat	3 weeks	Compared to baseline, in both groups ↘ HbA1c (*p* < .001), ↘ weight (*p* < .001), ↘ total body fat (*p* = .001), ↘ T‐cholesterol (*p* ≤ .001), ↘ LDL‐c (*p* ≤ .004), ↘ TAG in both groups (*p* ≤ .042), ↘ visceral adipose tissue (*p* = .024), (*p* < .001) for LCKD and LFD, respectively
11	Myette‐Côté et al. (2018)	*n* = 11 BMI 34.0 ± 8.0 kg/m^2^ Obese cases with type 2 diabetes	1. Low‐fat low glycemic index diet: ~55% CHO, 25% protein, 20% fat 2. Very low‐carb KD: <10% CHO, 25% protein, ~65% fat 3. Very low‐carb KD+ exercise: <10% CHO, 25% protein, ~65% fat +15 min of walking beginning 30 min after each meal	4 days	Compared to baseline in three groups: ↔ Glucose, ↔ triglyceride, and ↔ insulin level. Compared to baseline: ↘ proinsulin for VLCKD (*p* = .001) and VLCKD + exercise (*p* = .005), but ↔ proinsulin for low‐fat low glycemic index diet Compared to low‐fat low glycemic index diet ↘ mean glucose in the VLCKD with or without exercise (*p* ≤ .001), ↔ time in hypoglycemia
12	Romano et al. (2019)	*n* = 20 BMI 37.1 ± 6.8 kg/m^2^ Obese cases with type 2 diabetes	Low‐carb KD: 10%–20% of carbohydrate (≤25 g/day), 60%–70% protein, 25%–30% fat	2 weeks	↘ HbA1c (*p* < .0001), ↘ HOMA index (*p* < .0001), ↘ Weight (*p* < .001), ↘ BMI (*p* < .001), ↘ WC (*p* < .001), ↘ segmental (*p* < .001), whole fat mass (*p* < .001), ↘ AST (*p* < .0001), ↘ ALT (*p* < .0001)
13	Walton et al. (2019)	*n* = 11 BMI 36.3 ± 1.4 kg/m^2^ Obese cases with type 2 diabetes	Low‐carb KD: ~5% (≤30 g/day) of carbohydrate, ~20%–25% of protein, ~70%–75% of fat	13 weeks	↘HbA1c (*p* < .0001), ↘weight (*p* < .0001), ↗ HDL‐c (*p* < .005), ↘TAG (*p* < .005), ↘TAG: HDL‐c ratio (*p* < .005), ↔ LDL‐c ↔ AST, ↔ ALT
14	Albanese et al. (2019)	72 patients (60 F/12 M)	Very‐low‐calorie keto diet (VLCKD)	3 weeks	Total weight loss better in VLCKD than in VLCD group (5.8 ± 2.4 vs. 4.8 ± 2.5 kg, *p* = .008). Surgical outcomes: mean operative time slightly shorter in VLCKD group; percentage of patients requiring a longer‐than‐anticipated hospital stay lower in VLCKD group; lower drainage output and higher post‐operative hemoglobin levels in VLCKD group

Abbreviations: CONGA‐1, continuous overall net glycemic action of observations 1 h apart; CONGA‐4, continuous overall net glycemic action of observations 4 h apart. T‐Chol, total cholesterol; FBG, fasting blood glucose; HDL‐c, high‐density lipoprotein cholesterol; HOMA, Homeostasis Model Assessment; KD, ketogenic diet; LDL‐c, low‐density lipoprotein cholesterol; SU, sulfonylurea; T2D, type 2 diabetes mellitus; TAG, triacylglycerol; TMAGE, mean amplitude of glycemic excursion; WC, waist circumference.

The current review has detected that the HbA1c of diabetes under KD has been improved and even significantly reduced to 0.6%, 0.9%, and 1.3% in comparison to the control group based on the clinical trials. Furthermore, studies (Walton et al., 2019) and (Hussain et al., 2012) have revealed that diabetes under KD have experienced declined HbA1c levels from 8.9% to 5.6% (*p* < .0001) and from 7.8% to 6.3%, respectively. Moreover, researchers have declared that the maintenance of HbA1c effect could be achieved by long‐term counseling, with a significant reduction from 7.6% to 6.3% after 1 year, major changes observed in the first 70 days (Hallberg et al., 2018), or from 7.5% to 5.9% after 15 months (Webster et al., 2019), or from 6.6 to 6.1 for 12 months (Saslow, Daubenmier, et al., 2017) or from 7.2% to 6.3% for low carb versus 7.4% to 6.3% for high carb (*p* < .001) (Wycherley et al., 2016). The glycemic fluctuation looks to be maintained with a KD. A recent study by Tay et al. (2015) revealed a significant stability in the glycemic status in groups under low‐carb intervention; as a result, their blood sugar was majority fixed in the glycemic range (*p* = .07). KD has been also linked with significant normalization of fasting plasma glucose and mean blood sugar values, in both short‐period and long‐period clinical trials. Therefore, the studies are more coherent in relation to DM and weight loss. It can be seen that weight loss is more prevalent and more promised by KD and blood glucose is more controlled that can benefit DM patients.

Bariatric surgery (BS) as a way of reducing weight for severely obese patients is often conducted when other methods are failed to reduce weight such as diet, exercise, and drugs (Barrea et al., 2023; Zebari et al., 2022). However, KD can be used as a useful tool for preoperative weight reduction in bariatric surgery especially in short‐time conditions. For instance, a very‐low‐calorie KD can lead to considerable weight loss, and potential improvement in surgical risks before BS as well as reduction of hunger and feeling of rapid satiety (Colangeli et al., 2022). Furthermore, research concluded that patients conducted BS following very‐low‐calorie KD had better surgical outcomes in terms of lower stay at hospital, metabolic, and nutritional status that positively influenced tissue healing after bariatric surgery (Albanese et al., 2019). It can be understood that following KD can improve diabetes symptoms and blood glucose is better controlled. This is through a number of reasons mainly correlated with weight loss and reducing carbohydrate consumption.

#### 3.1.2 Cardiovascular disease and dyslipidemia

**FIGURE 2:**
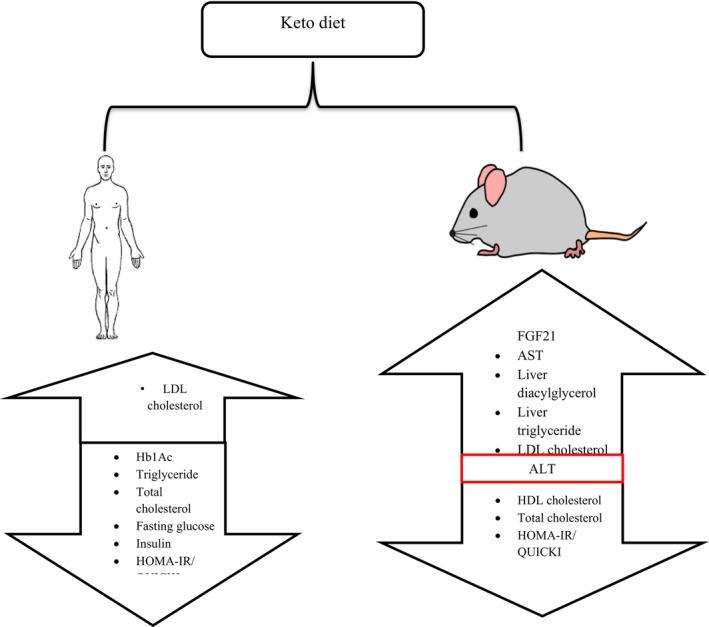
The impact of KD on lipid profile of two different models. In general, the studies have shown that in animal model total cholesterol and HDL and HOMA‐IR/QUICKI are decreased, no effect of ALT, and LDL AST, liver diacetylglycerol, and triglyceride increased. However, in human model except LDL, other biomarkers like Hb1Ac, tryglyceride, total cholesterol, fasting glucose, insulin, and HOMA‐IR/QUICKI decreased.

Cardiovascular disease is one of the most prevalent diseases in the world, and one of the main etiologies of cardiovascular disease is dyslipidemia (Malekpour et al., 2023). The impact of KD on some lipid profile and biomarkers is presented in Figure 2 (Jornayvaz & François, 2017). The authors stated that the differences in KD composition used in both models, it accounts for significant differences in results. High plant fat content is more positively affected but animal fat has negative effects.

Numerous studies have been conducted on both animal and human models with various study periods. For example, KD in animals for 2 weeks showed no significant effect on TG and fatty acid levels (Murata et al., 2013). In another study, the impacts of two types of KD were used on the fat level of mice; the first type was 78.7% fat and the other one was 92.8% fat; the researchers found that the latter one had lower high lipoprotein lipase (HDL) and higher triglyceride (TG) level but not significantly different and no results on low lipoprotein lipase (LDL) stated (Bielohuby et al., 2013). In another study, two groups of mice were fed with KD and standard mice diets for 5 weeks, the results found that LDL in both groups was similar (Jornayvaz et al., 2010). In a long‐term study (7–20 weeks), mice were fed with KD found to have twofold increase in their plasma TG and total cholesterol (Douris et al., 2015; Ellenbroek et al., 2014). However, in a 6‐week trial, mice fed with KD had lower total cholesterol and TG compared to other diets (Holland et al., 2016).

On the other hand, in human model, following KD had improved and caused physiological benefits such as improving HDL level (Brinkworth et al., 2009; Foster et al., 2003, 2010; Sharman et al., 2002; Tay et al., 2014), decreasing total cholesterol (Dashti et al., 2006), reducing TG (Dashti et al., 2006; Foster et al., 2010; Samaha et al., 2003; Tay et al., 2014), and decreasing LDL level (Dashti et al., 2006). In another study in 2012, it was found that KD led to decreased HDL, triglycerides, HbA1c, fasting plasma glucose, and increased HDL (Santos et al., 2012). In contrast, in some studies, LDL was increased significantly (Stern et al., 2004) and non‐significantly (Westman et al., 2008). In another research for 12 months, no significant differences were found in relation to serum cholesterol except in third month conventional diet had lower LDL level (Foster et al., 2003). It is interesting that the effect of KD on dyslipidemia might be related to ethnicity. In the study by Samaha et al. (2003), the authors found that Black individuals had lost less weight and higher TG compared to white subjects when consumed KD for 6 months with no significant difference in terms of HDL, LDL, and total cholesterol level.

Overall from the aforementioned studies, it can be understood that the impact of KD on dyslipidemia as a risk factor for cardiovascular disease in animal and human models is inconclusive. In the first case, it seems that lipid profile to be worsening and in latter one more improving. This difference can be attributed to the dissimilarity in terms of the KD composition used in both models. In animal model studies, mainly saturated fat‐rich diets are used, whereas in human models mainly unsaturated‐rich diet are followed.

It can be summarized that the results of both animal and human models depend on the KD component of the diet. Consuming higher saturated fatty acids might increase lipid profile particularly LDL. On opposite, diet low in saturated fat can improve lipid profile (HDL and TG and total cholesterol) and insulin level.

#### 3.1.3 Fatty liver (non‐alcoholic)

Non‐alcoholic fatty liver disease (NAFLD) is a non‐communicable and preventable disease that is known for hepatic adiposity potential development of inflammation, fibrosis, and cancer. So, it is considered one of the main chronic hepatic diseases. The initial stage of NAFLD is liver steatosis, wherein intrahepatic TG level exceeds 55 mg/g liver (Fabbrini et al., 2010). One of the main recommended dietary strategies to manage this case is weight loss. KD has been linked to address this problem in several studies, although sometimes might worsen the case due to high fat content and increased cholesterol levels.

A meta‐analysis study of 10 researchers concluded the impact of LCD on NAFLD stated that volunteers who followed LCD had significantly lower intrahepatic TG but no liver enzymatic changes (Haghighatdoost et al., 2016). Furthermore, KD can be more useful if applied than caloric restriction diet. A 2‐week clinical trial on the effect of LCD and low caloric diet on TG in NAFLD patients was conducted, and the results revealed that intrahepatic level of TG was greatly reduced in patients following LCD (Browning et al., 2011). Recently, in a very comprehensive review conducted on the impact of KD diet on NAFLD, the authors have concluded that the impacts of the KD on NALFD results are all positive but calories should be restricted in order to increase fat oxidation (Watanabe et al., 2020).

**FIGURE 3:**
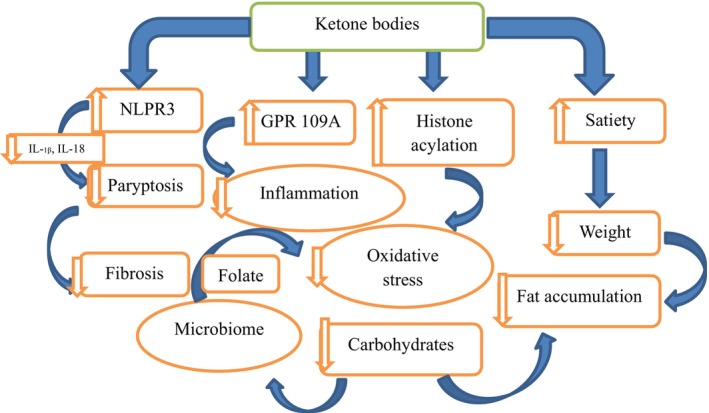
Elucidates the impact of KD non‐alcoholic fatty liver.

There are a few mechanisms, whereby the effect of KD may help with NAFLD (Figure 3). First, increase in fat oxidation as a result of low level of insulin, lipogenesis, and low carbohydrate consumption (Paoli et al., 2013). Second, food consumption control due to satiety induced by ketone bodies (Figure 3). Third, KD also leads to microbiome change and produces more folic acid as well as suppresses inflammation and oxidative stress (Mardinoglu et al., 2018). Histone acylation also believed to impart anti‐inflammatory traits. Another mechanism is GPR109A activation that possesses anti‐inflammatory traits, and NLRP3 inhibition plays a role in pro‐inflammatory cytokine activation including interleukins as well as fibrosis causing pyroptosis (Youm et al., 2015). The studies overall indicate that following KD can help reduce the NAFLD through restricting calorie consumption, weight loss, and increasing fat oxidation.

#### 3.1.4 Kidney failure and hypertension

Kidney failure and hypertension are alarmingly increasing in the community as a metabolic syndrome. Yet, there are a number of people who follow KD in order to control hypertension due to their problems in their kidneys. Furthermore, consuming relatively excessive amount of protein in KD is required to be taken into serious consideration. There are various animal and human and animal‐based model researches studying the impact of KD/LCD on the kidney and blood pressure (Table 2).

**TABLE 2:** The impact of some KD on kidney and hypertension.

No	References	Study duration	Experimental design	Diet types interventions	Outcome	Adherence level
1	Cicero et al. (2015)	1 year	An observational study with 377 Overweight and obese (BMI from 27 to 37) volunteers from 30 to 69 years old	KD (very low‐carb diet) composed of (carbohydrate 2–6 g per diet), proteins, 1.2/1.5 g/kg and low fat	Significant (*p* < .001) Diastolic BP (2.2–3.1 mmHg), and systolic BP (10.5–6.4 mmHg), changed from baseline to 3 months Beyond this time no more changes noticed	Up to 66 volunteers uncompleted the study not due to non‐clinical consequences
2	Bazzano et al. (2014)	1 year	A randomized control study of 148 obese‐free disease volunteers (BMI up to 45 kg/m^2^ aged from 22 to 75)	Low‐carb diet (less than 40 g/day) compared to low‐fat diet (30% fat with no more than 7% saturated fat)	By the end of the study mean, systolic BP differences were (3.60 mmHg) with no significant differences in diastolic and between groups	Volunteers were highly adhered to the study design
3	Liu et al. (2013)	1 year	A randomized controlled study of 50 female Chinese aged between 30 and 65 with normal BMI 24 kg/m^2^	Unlimited energy low‐carb (20 g/day) diet compared to low‐carb (20 g/day) energy‐limited diet	4.60 mmHg systolic BP and 2.70 diastolic mmHg difference seen by the end of the research. No impact on age groups	48 (96%) were highly adhered to the study design
4	Foster et al. (2010)	2 years	Low‐fat diet compared to low‐carb diet (20 g/day)	A randomized controlled study of 307 obese (32.6–39.6 kg/m^2^) patients aged between 36.8 and 55.2	By the end of the study 2.68, 3.19 mmHg difference for systolic and diastolic BP, respectively. No age group differences	68% adhered to low‐fat diet compared to 58% low‐carb diet
5	Lim et al. (2010)	2 years	A randomized controlled study of 133 patients from 37 to 57 years old with BMI 24 to 38 kg/m^2^ having one more CV risk factor	Control (no interventional diet). Very low‐fat diet versus high‐unsaturated diet versus very low‐carb diet (10%)	Significant BMI and diastolic BP difference (up to 5.2 mmHg) were seen in all the interventional groups compared to the control group	Moderate adherence stated
6	Yancy et al. (2010)	4 years	A randomized controlled study of 160 overweight (BMI > 25) and obese (BMI > 30) outpatient clinics	Low‐fat diet and Orlistat compared to low‐carb diet (less than 20 g/day)	Similar weight reduction noticed	No valid adherence evaluation
7	Gardner et al. (2007)	1.6 years	A randomized study of 311 pre‐menopausal overweight (BMI 27–40) female	Ornish diet (>10% energy from fat) versus LEARN diet (55%–60% energy from carbohydrate) versus Zone (40% carbohydrate) versus Atkins (carb <20 g/day after 3 months increased to 50 g/day)	Atkins diet group had the highest blood pressure throughout the study time. The decrease in (systolic 7.60 mmHg and diastolic 4.40 mmHg difference)	No valid adherence evaluation
8	Truby et al. (2006)	0.5 years	A randomized controlled study of 293 British volunteers	Atkins (carb <20 g/day) versus Rosemary Conley's diet versus slim‐fast versus WW diet	By the end of the study, Atkins diet led to reduction of systolic and diastolic BP reduction by 7.20 and 4.90. mmHg, respectively, but not statistically different from others	Adherence to each diet was dissimilar
9	Dansinger et al. (2005)	1 year	A single‐center randomized study of 160 Americans overweight and/or obese (mean BMI0 35) aged between 22 and 72 with having hyperglycemia, dyslipidemia, and hypertension	Atkins diet (carb <20 g/day), zone, Weight Watchers diet (calorie restriction) and Ornish diet (fat restriction)	No statistical differences in BP were seen among the diets	Low adherence score to the diets
10	Foster et al. (2003)	1 year	A randomized controlled study of 63 males and females obese	Conventional diet (high‐carb, low‐fat, low‐calories versus high‐protein diet) was compared to low‐carb diet (20 g/day)	Some reduction in systolic BP (2.7 mmHg) and diastolic BP (0.1 mmHg) seen but were not significantly different	Level of adherence of both considered low
11	Samaha et al. (2003)	0.5 year	A randomized control study of 132 obese individuals (BMI ≥ 35)	Low‐fat diet compared to low‐carb diet (carb <30 g/day)	Some reduction in systolic BP (1.0 mmHg) and diastolic BP (3.0 mmHg) seen but were not significantly different	Higher adherence reported in low‐carb group

Abbreviations: BMI, body mass index; BP, blood pressure.

A previously published systematic review about the impact of very LC diet on normal kidney function reported that the impact was very scarce (Rolland et al., 2013). In an animal model, it was found that diet induces ketosis ameliorates renal cyst in polycystic kidney disorder (Torres et al., 2019). In human, KD is linked with decrement of both blood systolic and diastolic pressures (Samaha et al., 2003). A 12‐month study showed a better improvement in blood pressure (both blood systolic and diastolic) after following KD compared to following low‐fat diet by using orlistat (Mayer et al., 2014). Furthermore, a study showed systolic pressure improvement after following 3 months of KD, beyond this period until a year no other improvement was noticed (Cicero et al., 2015).

Although data on the effect of LCD diet on the renal function are relatively limited, it is worth shedding light on particularly in the long term. One of the most widely expressed concerns is kidney stone development which is noticed in data on pediatric epilepsy (McNally et al., 2009). There is no a conclusive guideline in relation to protein consumption. But it is recommended between 0.8 (Ko et al., 2017) and 1.4 g/body kg (Bakris, 2005). Taking excessive amounts of animal protein might induce and promote kidney stones (Tracy et al., 2014) particularly red meat is harmful, but it is dose dependent (Lew et al., 2017); other sources including dairy products, eggs, and seafood are less detrimental and plant‐based proteins potentially protective (Chauveau & Lasseur, 2019). Another factor is acidosis which might affect calcium availability. Furthermore, taking high amount of fat and protein and low fruits and vegetables as practiced by Western diet promoted the risks of albuminuria (Lin et al., 2010). In a study among 12,000 participants for 23 years, it was observed that high protein consumption was linked to the 23% increased risk of CKD. Other studies have observed similar results (Lew et al., 2017; Mirmiran et al., 2020).

KD in patients with CRD can be of particular concern because of high protein content. It has been reported that those who daily consume high protein content of more than 1.5 g/kg might compromise glomerular filtration rate (Ko et al., 2020). Furthermore, consuming high protein develops hyperfiltration, a health condition when blood flow to glomerulus increases and damages them (Kim & Jung, 2020). Moreover, KD diet may increase ketone body and acidosis and consequently worsen kidney diseases in CRD (Banerjee et al., 2015). A case report stated that a 6‐year decline in renal function was improved by changing high‐carbohydrate diet to low‐carbohydrate and high‐fat diet (Nielsen et al., 2006). Thus, the physiological benefits of KD in patients with blood pressure and kidney failure are controlled by the KD composition and the duration. The results indicated that patients with kidney problems should be cautious using KD. It is particularly important to be supervised under nutritional expertise in order to avoid negative consequences.

#### 3.1.5 Polycystic ovary syndrome

Polycystic ovary (PCOS) is female metabolic and reproductive system syndrome. The prevalence of the disease is up to 10% globally and represents up to 70% of infertility in women of reproductive age (Walters et al., 2018). So far, the exact mechanism and etiology of the PCOS is not fully understood; however, several mechanisms have been proposed including heredity and environmental causes such as unhealthy dietary habit, low childbirthweight, physically inactive, obesity, and sleep apnea (Patel, 2018).

Recent dietary intervention has been suggested to clinically reduce the symptoms of PCOS including hormonal imbalance, abnormal menstruation, and ovulation. Thus, dietary behavior changes play a significant role in ameliorating the clinical signs and symptoms of PCOS.

LCD has been documented to effectively lose weight and help with management of infertility in obese individuals (Goss et al., 2014; Marsh et al., 2010). Very earlier studies showed low‐carbohydrate KD restored LH/FSH ratio and testosterone level, insulin, and weight in PCOS (Mavropoulos et al., 2005). In a meta‐analysis of randomized controlled trials, the data showed that the LCD can be one of the crucial interventions to improve the clinical symptoms of PCOS (Zhang et al., 2019). A pilot study of 11 women (BMI more than 27) who were clinically diagnosed with PCOS showed that following KD improved weight management and infertility over 6‐month study (Mavropoulos et al., 2005). Earlier studies also stated that following LCD was significantly improved abnormalities in their reproductive system (Stamets et al., 2004). Recently, a study of 17 obese women with PCOS was registered for diet intervention; the authors have concluded that KD had positive effect on PCOS results in a short period of time (Cincione et al., 2021). In the same year, another group of researchers studied the impact of LCD on PCOS in women. They found that the diet was effective and feasible to positively influence control glycemia and weight in PCOS (Missel et al., 2021). In more recent study, Mediterranean diet with LC is affective in restoring menstrual cycle and hormonal level in PCOS patients suggested for the treatment of overweight women with PCOS (Mei et al., 2022). Thus, the studies all refer that LCD and KD can be a suitable nutritional intervention to mitigate PCOS symptoms.

Several mechanisms have been suggested by which LCD improves reproductive abnormalities in PCOS patients. LCD improves insulin resistance. It is well documented that hyperinsulinemia and insulin resistance is a crucial factor in the progress of hyperandrogenism, abnormal metabolism, and menstrual cycle and anovulation in both obese and non‐obese PCOS patients (Kujawska‐Luczak et al., 2018). Hyperandrogenism is caused by hyperinsulinemia which induced adrenal and ovarian glands to produce more androgen and limit hepatic production of SHBG. Furthermore, hyperinsulinemia also affects reproductive system by limiting follicular progression and fertility (Bates & Legro, 2013; Faghfoori et al., 2017). LCB can also restore carbohydrate metabolism, ovarian dysfunction, and gonadotropin abnormalities in PCOS through inositol metabolism which is cyclohexanol stereoisomer related to vitamin B complex family member. The imbalance between the two inositols such as myoinositol and D‐chiro‐inositol can cause insulin resistance. Thus, inositol metabolism misregulation can cause hyperinsulinemia and ameliorate insulin sensitivity, follicles, and PCOS development (Laganà et al., 2018). Consequently, LCD can reset inositol balance and as a result leads to decrease androgen level, increase insulin resistance, restore menstrual cycle, and improve infertility and oocytes in PCOS patients. The results indicated that the KD and\or LCD can be an effective way to reduce the symptoms of PCOS. This is because KD possesses an affective impact on controlling insulin level in the body.

### 3.2 Non‐metabolic diseases

#### 3.2.1 Oral health

The progress of dental cavities majorly depends on the fermentable carbohydrates. Similarly, restricting carbohydrates is believed to reduce the chances of inflammation of gingival which is periodontal disease prerequisite (Nyvad & Takahashi, 2020). Moreover, although there are not enough clinical data regarding KD and oral health, however, there is a growing evidence suggesting that restricting simple and processed carbohydrate can greatly reduce the possible inflammation of gingival (Woelber et al., 2019).

There are very little data available about the impact of KD/LCD on oral health. However, a few studies highlighted these effects. In 6‐week non‐controlled trial study, the influence of KD on oral health was studied. The authors found no significant differences in any measured parameters. However, the results cannot be generalized because the participants were healthy and had a proper oral hygiene (Woelber et al., 2021). LCD rich in nutrients significantly decreases gingival inflammation and periodontal disease. In another study, 10 volunteers were put on Paleo diet that encourages low carbohydrate consumption for 4 weeks without accessing to oral hygiene; the researchers found that mean bleeding reduced from 34.8% to 12.6% and periodontal pocket depths improved to 0.2 mm. It is believed that this is because of not consuming simple sugar (Baumgartner et al., 2009).

There are a few mechanisms that carbohydrate might change the gingival inflammation. It has been reported that limit of carbohydrate consumption limits teeth erosion by acid. Also, higher consumption of sugar leads to microbial imbalance and inflammation and restricts the periodontal ligament cell proliferation. Furthermore, low‐carbohydrate diet can decrease caries, formation of calculus, and gingivitis by more than half (Woelber et al., 2016). However, more research is needed to confirm or refute the benefits or detrimental effects of KD/LCD on oral health. The overall results refereed that the KD can be an appropriate way to reduce oral problems; this is through reducing simple and quickly digested and fermented carbohydrates.

#### 3.2.2 Libido and sex drive

Studies have shown that diet has a direct relationship with health, and its influence is undeniable (Katz & Meller, 2014). Sex drive is part of one's health that can be affected by diet. For instance, research in this regard has shown that unhealthy diets might lead to different metabolic syndromes such as diabetes and low level of human sex hormone regulator: testosterone (La et al., 2018). It has been reported that high amount of docosapentaenoic acid intake found mainly in fish decreased females’ risk of an ovulation (Mumford et al., 2016).

There are not many studies on some diets like KD on libido. However, a few studies have shown that calorie restriction and weight loss can have positive impact on libido (Khoo et al., 2010). In a study on obese female individuals, it has been reported that ketosis significantly improved general women's sexual ability (Castro et al., 2018). On the other hand, to compare low fat and LCD for weight loss, the results indicated that out of 29 only 1 participant experienced low sex drive (LeCheminant et al., 2007). A study compared KD and Western diet in relation to sex drive, the outcomes referred that in KD group the level of testosterone was higher (Silva, 2014). A recent study found that KD improved testosterone level (Wilson et al., 2020). In another study, KD group not just had better testosterone level but also had better function of testicular (Mongioì et al., 2020). Studies also reported the association between KD and improvement of sexual drive and functions (Dhatariya, 2016; McDonald & Cervenka, 2019). In a very recent study, obese and diabetic adults with chronic diseases experienced better sexual function. However, in another study, free serum testosterone increased, and attached testosterone to protein was increased (Abboud et al., 2021).

The mechanism of KD affecting libido is now comprehensively known; however, some factors have been linked to that. First, KD can reduce blood glucose and insulin level, re‐balance hormonal level in women, and reduce risks of PCOS, thus leading to more favorable sexual drive. Second, KD can improve mood and address its related issues including low level of serotonin, deficiency of gamma‐aminobutyric acid (GABA), mitochondrial dysfunction, insulin resistance, and inflammation (Brietzke et al., 2018). Despite that, KD might temporarily slow down libido due to some side effects of low carb consumption rather than the negative impact of KD on libido. Moreover, previous studies have shown that KD can address problems associated with low level of serotonin, GABA, inflammation, oxidative stress, and insulin resistance (Brietzke et al., 2018). Therefore, the success that one can get from this diet is to improve mood and increase self‐confidence. The results might conclude that KD can reduce libido at the beginning due to side effects, but once the person adapts to the diet it can boost sex drive through losing weight and gaining more confidence.

#### 3.2.3 Mental and psychiatric problem

It is common understanding that KD was first used for the treatment of epilepsy. Today, several studies have shown that KD can be used as a technique to reduce mental and psychiatric problems. The available data and evidence show the intervention of KD for several mental and psychiatric disorders including Alzheimer, anorexia nervosa, autistic spectrum disorder (ASD), bipolar disorder, major depressive disorder, schizophrenia, and narcolepsy (Table 3). The data have shown that KD can stabilize mood, decrease anxiety, and several other mental and psychiatric parameters. However, the benefits remain on level of inheritance to the diet.

**TABLE 3:** The impact of KD on some mental and psychiatric disorders.

No	Reference	Problem type	Characteristics	Experiment design	Outcomes
1	Morrill and Gibas (2019)	Alzheimer	A case report during 10‐week treatment	High fat and low carb to generate 0.5–2 mg/dL ketone	MoCA score improved from 21 to 28
2	Taylor et al. (2018)	Alzheimer	Pilot study of 17 volunteers for 31 months	KD composed of 70%, 20%, and 10% from fat, protein, and carb, respectively	ADAS‐Cog and MMSE scores significantly improved
3	Ota et al. (2019)	Alzheimer	RCS for 12 weeks of 20 patients	KD composed from MCT	WAISIII and WMS‐R were significantly improved in the last week
4	Scolnick et al. (2020)	Anorexia Nervosa	A case report for 6 months	Diet composed of 1:1 to 2:1 ratio of carbohydrates to fats	Symptoms were gone (limit of eating obsession), and further improvement was seen beyond 6 months
5	Lee et al. (2018)	Autistic disorder	Six months CT for 15 patients	Modified KD with MCT	ADOS‐2 was improved at 3 and 6 months among 66% of the participants
6	Evangeliou et al. (2003)	Autistic disorder	Pilot study of 30 participants for 6 months	KD with MCT	Little to significant CARS improvement was seen among 60% of the participants
7	Herbert and Buckley (2013)	Autistic disorder	A case report for 8 years	Gluten‐free KD	CARS score was improved from 49 to 17 and quotient of intelligence was developed by 70%
8	Żarnowska et al. (2018)	Autistic disorder	A case report of 6‐month diet following 10‐month observation	Standard KD	WISC‐R was improved by the end of the study
9	El‐Rashidy et al. (2017)	Autistic disorder	Case–control of 45 patients for 6 months	Atkins and gluten and casein free diet comparing to standard control	ATEC score was significantly reduced from Atkins diet (58–44) gluten free (64.13–42.13) CARS score was significantly improved by the Atkins diet (41.7–33.7) and gluten free (39.17–34.27)
10	Campbell and Campbell and Background (2019)	Bipolar	Observational study of 141 patients	Standard KD enriched with omega 3	KD significantly improved mood stabilization
11	Phelps et al. (2013)	Bipolar	Two cases of study for 7 months	Standard KD	Improvement of mood was obtained and further improvement seen with medication
		Bipolar	Observational study of 272 patients		
12	Cox et al. (2019)	MDD	A case report for 12 weeks	10% carbs, 25% protein, and 65% fat	PHQ‐9 score was improved
		MDD	A case report for 4 weeks	Very low KD	Reduce anxiety and mood stabilized
13	Husain et al. (2004)	Narcolepsy	CT of 9 patients for 9 participants	KD with carb less than 20%	NSSQ score was improved by 18%
14	Kraft and Westman (2009)	Schizophrenia	A case report for 12 weeks	KD with carb less than 20%	Visual and auditory hallucinations were controlled. Further improvements were seen after 1 year
	Palmer (2017)	Schizophrenia	Two case reports: one for 8 weeks and the other one for 36 weeks	Standard KD	Mood improved and delusion and hallucination were reduced
	Saraga et al. (2020)	Bipolar disorder	A case report/no duration mentioned	Modified KD. Ratio of carbohydrate, protein and fat (3 + 1 + 2 g)	Better depression and manic symptoms
	Chmiel (2022)	Bipolar disorder	A case report for 1 year	KD with 5% carbs, 15% protein, and 80% fat	Better sleep, mood stabilization, cognitive function, and less anxiety
15	Danan et al. (2022)	Bipolar disorder, Schizophrenia, and depressive disorder	Retrospective cohort study of 31 for mean 1–34 weeks	KD with carb less than 20 g/day	Severity and clinical symptoms of psychosis and depression significantly improved

Abbreviations: ADAS‐cog, Alzheimer's disease assessment scale‐Cognitive Subscale; CARS, Childhood Autism Rating Scale; CT, clinical trial; MCT, medium‐chain triglycerides; MDD, major depressive disorder; MMSE, Mini‐Mental State Examination; MoCA, Montreal Cognitive Assessment; NSSQ, Narcolepsy Symptom Status Questionnaire; PHQ‐9, Patient Health Questionnaire; WAIS, Wechsler Adult Intelligence Scale; WMS‐R, Wechsler Memory Scale.

Studies proposed a number of mechanisms by which KD attenuates mental and psychiatric disorder symptoms. It is deemed that KD modifies GABA to glutamate, changes and balances the level of GABA, and consequently reduces delusions and hallucinations in schizophrenia patients (Włodarczyk et al., 2018). Furthermore, KD might decrease oxygen‐reactive species and inflammation in the brain which consequently attenuates the symptoms of brain‐related diseases including Alzheimer (Gasior et al., 2006). Moreover, KD controls apoptosis and hence neuronal excitability and reduces frequency of epilepsy (Ułamek‐Kozioł et al., 2019). Several metabolic alterations related to epilepsy can also benefit ASD via positive modifications in the microbial community in the gut (Lee et al., 2018). Additionally, KD can acidify the plasma and decrease intracellular calcium and sodium that is believed to stabilize mood in patients with bipolar disorder (Phelps et al., 2013). Finally, KD is known to induce hypoglycemia and activate orexin‐containing neurons. Hence, it improves the sleepiness in patients with narcolepsy (Husain et al., 2004). The results have shown more coherent evidence about the benefits of KD for the mental problem particularly epilepsy and stabilizing mood.

#### 3.2.4 Osteoporosis and skeletal system

The influence of KD on bone in animal models is a bit different. In a study feeding mice for KD for 3 months, it has been reported that KD led to substantial bone density loss, and decreased appendicular bone biochemical role but less influence on axial bone (Ding et al., 2019; Saito et al., 2016). In animal studies, ultrastructure of micro‐CT showed that at the dose of 100 mg/kg, KB showed significantly affected osteoporosis prevention by reducing concentration of calcium as an indirect indicator of reduced UBR activity and increased bone volume in ratio to total ratio of volume (BV/TRV) (Davie et al., 1986). It is not surprising that diet including KD has a direct impact on the body's health including skeletal structure and bones. In vitro data have shown that osteoblast (OBL) known as unit of bone remodeling (UBR), alkaline phosphate, and bone mineral content (BMC) are revealed to be controlled by the types of predominant ketone bodies (KB). Furthermore, the mineralization activity of OBL is known to be down‐regulated by 3‐β‐hydroxybutyrate (3βOHB) and up‐regulated by acetoacetate.

In animal studies, it also has been stated that cortical and abnormal cancellous and low BMD were detected under KD experiment (Wu et al., 2017) (Table 4). A more up‐to‐date study, every other day KD (EODKD), and a combination of intermittent fasting and KD as a novel nutritional intervention for epileptic individuals, but the effects were not understood clearly (Hartman et al., 2013). It was seen that EODKD generated more ketones than KD in cerebrospinal and serum fluid (Wang et al., 2017). In more recent study, the authors compared different KDs using a number of rats groups, standard diet, intermittent KD, and continuous KD. After 12 weeks of treatment, body fat percentage and bone BMD were measured by DEXA, and micro‐CT was used to assess bone mechanical and microstructure properties and some other bone parameters. The results showed that both KD and EODKD had higher fat percentages and ketone levels but less body weight compared to the control. On the other hand, both KD and EOKDK compromised both mechanical properties and bone mass. So, EODKD increased ketosis but did not have any detrimental effect on mechanical or bone strength (Xu et al., 2019). However, other studies showed that KD results in less bone stiffness and strength (Ding et al., 2019). Similarly, a study showed that using different standards and KDs in mice, KD diet has caused significant bone loss (Wu et al., 2019) (Table 4). Therefore, it can be understood that the effect of KD in human and animal models is different depending on the KD composition, experimental experiment design, and duration.

**TABLE 4:** The impact of KD on skeletal system of human–animal models.

No	Resource	Study type/duration	Experimental design	Assessment	Criteria assessed	Outcome
1	Bergqvist et al. (2008)	Clinical/1 year and 3 months	Fifteen children in the US having sporadic epilepsy	The volunteers were given keto diet based on weight 1:4 (CHO and Protein: Fat)	Bone growth, health nutritional status and seizure, DEXA assessed body, and LC‐BM Z‐score	KD led to poor health in lower BMI children and more BM loss
2	Davie et al. (1986)	Observational study/4 weeks	Eight obese British	Subjects were given low calorie (3000 ± 300 kJ/day) and high dose of calcium (32 ± 3 mmol/day) diet	Impact of diet as well as tri‐iodothyronine on zinc, phosphate and calcium, zinc balances, and hydroxyproline in urine	Statistically higher calcium retained when CHO is increased, but balance of calcium was generally positive
3	Andersen et al. (1997)	Randomized controlled trials/2 years	Twenty‐one healthy obese US female	Two groups were gone on diet and diet with training. The diet included 925 kcal/day diet for the first 17 weeks and calories increased to 1000–1500 kcal for the rest of the study	DEXA (BMD) and BM of femoral neck (FN) and greater trochanter (GT) free fat mass and fat mass prior and post 24 weeks study	No difference between the two diets. Loss of BMD might be prevented but not in FN and GT, by increasing energy content of 925 kcal/day
4	Bonjour et al. (1991)	Prospective cohort study/2 years	Forty‐five children (6.6 mean age) with refractory epilepsy put on KD	Standard KD (90% calories from fat and 10% from protein and CHO)	Growth factors (weight, height, and body mass)	9% deceleration of growth, but nutritional status maintained and improved
5	Bonjour et al. (1991)	Prospective and longitudinal study/6 months	Sixty‐three patient	Standard KD	Baseline and 6‐month interval, right TH DEXA, LS; Serum 25 OHD, PTH, OC, phosphate, ALP, calcium, urinary creatinine/calcium ratio	Trends of LS BMD decrease annually. 68% decrease of *Z*‐score BMD in 6‐month study, moveable patients had higher bone loss. Increased urinary creatinine/calcium ratio. One patient progressed renal calculus
6	Neal et al. (2008)	Prospective and longitudinal study/up to 1 year	Seventy‐five British children	Volunteers put on two standard KD (classical medium‐chain triglyceride (MCT))	Growth pattern, height, weight, BMI *Z*‐score at measured 3 months interval up to a year	By 6 and 12 months *Z*‐score reduced especially in youngsters, weigh reduced in MCT group at 3 months, MCT had higher protein intake but nor changes in height, weight, and BMI between the two diet groups
7	Shum et al. (2016)	Controlled study/3 months	Thirty US (50% control: 50% patients)	Controls group no restriction diet. Other group consumed CHO <20 g/day for the first month, then <40 g/day of CHO for the rest of the study	Weight loss, bone turnover, primary end point at 3rd month and secondary end point at 1st month	Weight loss more significant in low CHO diet, no bone turnover differences
8	Hammond et al. (2019)	Clinical trial	Nine British runners aged around 21 ± 2 conducting high‐intensity running protocol at morning and afternoon under special diet planning including high CHO, low CHO, high‐fat diet, low CHO, and low energy		The impact of post‐running CHO and energy limitation on change of cell signaling pathway Muscle and biomarkers of metabolism of bone	BCTX showed significant reduction in high CHO diet in comparison to other types of the diets
9	Bielohuby et al. (2010)	CT	24 male rats (Wistar)	Given different diets: HF‐LC I (fat = 94.5%, protein = 4.2%, and carbohydrate = 1.3%), HF‐LC II (fat = 66%, protein = 33%, and carbohydrate = 1%), normal diet (fat = 9%, protein = 33%, and carbohydrate = 58%)	Humeri histology, RT‐PCR of bone marrow, 25OHD, CTX, GH, IGF‐1, Leptin OC. Serum calcium and phosphate and rat PTH. pQCT μCT assessment	HF‐LC II associated with decreased tibia, femur, and humerus length pQCT μCT assessment showed noticeable decrease BMD of tibia and maximum load of HF groups as well as serum PINP1. Furthermore, RT‐PCR revealed reduction of bone marrow expression Runx2 and osterix (70–80)
10	Wu et al. (2017)	Controlled trial	Eight‐week‐old mice for 12 weeks	The standard diet with keto diet	Middle femur cortical and distal femur trabecular bone CT scanning	The cortical and cancellous to long bones were compromised by KD and more bone loss
11	Hartman et al. (2013)	Controlled trial	Sprague –Dawley rats	Standard diet, EODKD, intermittent KD, and KD	Fat percentage BMD by Dexa, micro‐CT analysis of mechanical and microstructure of bone	EODKD showed higher ketone than KD, osteoclastic pathway inhibited and initial differentiation of osteogenic
12	Ding et al. (2019)	Controlled trial	Sprague–Dawley	Standard and KD for 12 weeks	Trabecular and cortical bone by micro‐CT, stiffness, and strength of bone calculation by microfinite analysis	KD cause more bone loss and less biomechanical function on appendicular
13	Bielohuby et al. (2010)	Controlled trial	Twenty‐four Wister rats	Standard diet, low‐carb high‐fat diet I (66% fat, 33% protein, and 1% carbohydrate), low‐carb high‐fat diet II (94.5 fat, protein 4.2%, and 1.3 carbohydrate)	pQCT, bone marrow, insulin‐like growth factor protein, Leptin serum phosphate, and calcium	Low‐carb high‐fat diet I associated with more bone marrow and visceral fat, increased Leptin Low‐carb high‐fat diet II decreased tibia, femur length, and humerus pQCT showed reduction in BMD tibia in KD groups

Abbreviations: 250HD, 25 OH vitamin D; PTH, parathyroid hormone; BCTX, carboxyl terminal collagen crosslinks; CT, clinical trial; FN, femoral neck; GT, greater trochanter; ND, not determined; PlNP, procollagen; pQCT, peripheral quantitative computed tomography; RT‐PCR, real‐time PCR.

In both human and animal models, the effect of KD or LCD on osteoporosis and skeletal system has been investigated. The results seem to be inconclusive not just between the two models but also among human models. For instance, in very earlier research, it was found that the epileptic children following KD and using anticonvulsant drugs for a year caused bone mass density (BMD); however, it could be reversed by taking Vit D supplements (Hahn et al., 1979). In a short‐term study of 6 months of KD among seven volunteers, the researchers did not find any change in bone mineral content (BMC) (Bertoli et al., 2002). After that, in a more comprehensive study, the influence of KD on 25 epileptic children, it was reported that the children were diagnosed with lower BMC baseline (age standardized) and lumbar spine decreased by 0.6 SD annually and *Z*‐score referred to positive BMC based on age and BMI (Bergqvist et al., 2008) (Table 4). Furthermore, BMC was seen to decline when children followed KD. In contrast, in a 5‐year study of KD in Glucose Transporter 1‐deficit patients, no negative effect on BMC was seen in both baseline and during the diet (Sheth et al., 2008). In another human model of 66 overweight and obese volunteers to compare LCD and isocaloric low‐fat diet, the results showed no significant differences between the two diets (Brinkworth et al., 2016). But a more recent study found a decrease in bone markers in athletes who followed KD for nearly a month (Heikura et al., 2020). Based on the above‐mentioned studies, it can be summarized that KD has more profound effects on children than adults. This can be attributed to the impact of KD on the skeletal development stages. Therefore, health professional bodies and International KD study group consensus recommend close monitoring, particularly using Dexa of children during KD treatment (Bertoli et al., 2014; Simm et al., 2017).

## 4 CONCLUSION

This comprehensive review summarizes the impact of KD on some metabolic and non‐metabolic diseases. It is obvious that KD has caused and still ongoing debate. The studies show that KD could be useful in reducing mental and psychiatric problems and cause more stable mood and less anxiety. It can also be useful in reducing glucose and insulin resistance. Similar to that it helps with sex drive. Also in both short‐ and long‐term keto diets, it can improve hunger, fat oxidation, and weight loss. KD can also help PCOS by balancing hormones and insulin resistance. However, it is duration and KD fat‐type dependent. However, the impact of KD on cardiovascular diseases, kidney and hypertension, non‐alcoholic fatty liver, and skeletal structure needs more studies, and there is no robust evidence. Some show negative impact and some show significant effects due to difference in animal and human model, and it is dose and duration dependent.

## AUTHOR CONTRIBUTIONS

**Yaseen Galali:** Project administration (equal); writing – original draft (equal). **Salih M. S. Zebari:** Conceptualization (equal). **Ahmed Aj. Jabbar:** Software (equal). **Holem Hahm Balaky:** Writing – review and editing (equal). **Bashdar Abuzed Sadee:** Resources (equal). **Hamed Hassanzadeh:** Supervision (equal); validation (equal).

## FUNDING INFORMATION

There is no financial support associated with this work.

## CONFLICT OF INTEREST STATEMENT

There is no conflict of interest to declare.

## Data Availability

Data sharing is not applicable to this article as no new data were created or analyzed in this study.
